# Biocompatible Poly(ε-Caprolactone) Nanocapsules Enhance the Bioavailability, Antibacterial, and Immunomodulatory Activities of Curcumin

**DOI:** 10.3390/ijms251910692

**Published:** 2024-10-04

**Authors:** Floriana D’Angeli, Giuseppe Granata, Ivana Roberta Romano, Alfio Distefano, Debora Lo Furno, Antonella Spila, Mariantonietta Leo, Chiara Miele, Dania Ramadan, Patrizia Ferroni, Giovanni Li Volti, Paolo Accardo, Corrada Geraci, Fiorella Guadagni, Carlo Genovese

**Affiliations:** 1 Department of Promotion of Human Sciences and Quality of Life, San Raffaele Roma Open University, 00166 Rome, Italy; antonella.spila@sanraffaele.it (A.S.); mariantonietta.leo@uniroma5.it (M.L.); chiara.miele@sanraffaele.it (C.M.); dania.ramadan@uniroma5.it (D.R.); patrizia.ferroni@uniroma5.it (P.F.); fiorella.guadagni@uniroma5.it (F.G.); 2 CNR-Institute of Biomolecular Chemistry, Via Paolo Gaifami 18, 95126 Catania, Italy; giuseppe.granata@icb.cnr.it (G.G.); paolo.accardo@yahoo.it (P.A.); corrada.geraci@icb.cnr.it (C.G.); 3 Department of Biomedical and Biotechnological Sciences, Section of Physiology, University of Catania, 95123 Catania, Italy; ivanarobertaromano@yahoo.it (I.R.R.); lofurno@unict.it (D.L.F.); 4 Department of Biomedical and Biotechnological Sciences, Section of Biochemistry, University of Catania, 95123 Catania, Italy; distalfio@gmail.com (A.D.); livolti@unict.it (G.L.V.); 5 InterInstitutional Multidisciplinary Biobank (BioBIM), IRCCS San Raffaele, 00166 Rome, Italy; 6 Department of Medicine and Surgery, “Kore” University of Enna, Contrada Santa Panasia, 94100 Enna, Italy; carlo.genovese@unikore.it; 7 Nacture S.r.l, Spin-Off University of Catania, Via Santa Sofia 97, 95123 Catania, Italy

**Keywords:** poly(ε-caprolactone) nanocapsules, curcumin, bioaccessibility, antibacterial activity, probiotics, inflammation, ADSCs, LPS, cytokines, immunomodulatory activity

## Abstract

Curcumin (Cur), the primary curcuminoid found in *Curcuma longa* L., has garnered significant attention for its potential anti-inflammatory and antibacterial properties. However, its hydrophobic nature significantly limits its bioavailability. Additionally, adipose-derived stem cells (ADSCs) possess immunomodulatory properties, making them useful for treating inflammatory and autoimmune conditions. This study aims to verify the efficacy of poly(ε-caprolactone) nanocapsules (NCs) in improving Cur’s bioavailability, antibacterial, and immunomodulatory activities. The Cur-loaded nanocapsules (Cur-NCs) were characterized for their physicochemical properties (particle size, polydispersity index, Zeta potential, and encapsulation efficiency) and stability over time. A digestion test simulated the behavior of Cur-NCs in the gastrointestinal tract. Micellar phase analyses evaluated the Cur-NCs’ bioaccessibility. The antibacterial activity of free Cur, NCs, and Cur-NCs against various Gram-positive and Gram-negative strains was determined using the microdilution method. ADSC viability, treated with Cur-NCs and Cur-NCs in the presence or absence of lipopolysaccharide, was analyzed using the 3-[4,5-dimethylthiazol-2-yl]-2,5-diphenyl tetrazolium bromide assay. Additionally, ADSC survival was assessed through the Muse apoptotic assay. The expression of both pro-inflammatory (interleukin-1β and tumor necrosis factor-α) and anti-inflammatory (IL-10 and transforming growth factor-β) cytokines on ADSCs was evaluated by real-time polymerase chain reaction. The results demonstrated high stability post-gastric digestion of Cur-NCs and elevated bioaccessibility of Cur post-intestinal digestion. Moreover, Cur-NCs exhibited antibacterial activity against *Escherichia coli* without affecting *Lactobacillus* growth. No significant changes in the viability and survival of ADSCs were observed under the experimental conditions. Finally, Cur-NCs modulated the expression of both pro- and anti-inflammatory cytokines in ADSCs exposed to inflammatory stimuli. Collectively, these findings highlight the potential of Cur-NCs to enhance Cur’s bioavailability and therapeutic efficacy, particularly in cell-based treatments for inflammatory diseases and intestinal dysbiosis.

## 1. Introduction

Inflammation is a defense mechanism through which our body can neutralize harmful agents overcoming host anatomic barriers. Among them, it is possible to distinguish pathogens and related virulence factors (e.g., toxins, capsules, and biofilm), damaged or infected cells, chemicals, and irradiation [1]. Microorganisms invasion is recognized through the pattern recognition receptors (PRRs) (e.g., Toll-like receptors) present on the membrane of innate immune cells, such as macrophages [2]. Consequently, such cells release soluble mediators known as cytokines. These are low-molecular-weight proteins, able to interact with specific receptors, triggering cellular events that can promote or suppress the inflammatory response [3,4]. Inflammatory status modulation can be realized thanks to the availability of many cytokines, which can be generally classified as pro- and anti-inflammatory. The first group comprises a series of molecules, including tumor necrosis factor-α (TNF-α) and interleukin-1β (IL-1β). Such molecules can be produced and released by immune system cells, including macrophages, activated lymphocytes, endothelial cells, and fibroblasts [4]. The anti-inflammatory cytokines include interleukin-10 (IL-10) and the transforming growth factor-β (TGF-β), two distinct molecules that allow the inflammatory process to extinguish. Indeed, these molecules favor tissue repair, by restoring extracellular matrix and vascular permeability to normal conditions [5]. Thus, inflammation is a protective response essential to ensure the integrity of the organism, and it is destined to resolve upon removing the causing insult. However, in some cases, the inflammatory state can persist, thus evolving into a chronic condition [6].

The main problem associated with chronic inflammation is the persistent activation of the immune system, which can favor the onset of a large variety of diseases [7], including diabetes [8], cancer [9], neurodegenerative [10,11] and cardiovascular [12] diseases, and autoimmune disorders [13]. Accordingly, the ability to interfere with the immune functions and to affect the release of pro-inflammatory cytokines are biological properties essential to avoid prolonged inflammation.

In this regard, it has been shown that human adipose-derived stem cells (ADSCs) exert immunomodulatory activity, playing a protective role in different inflammatory contexts [14,15,16]. Recently, in an in vitro model of diabetic retinopathy, we proved the ability of ADSCs to modulate the release of inflammatory cytokines such as IL-1β and TNF-α from human retinal endothelial cells exposed to high glucose levels [17,18]. A further study showed that ADSCs prevented non-alcoholic steatohepatitis-related fibrosis, downregulating the pro-inflammatory IL-17 [19].

Besides stem cells, natural compounds can also exert immunomodulatory activity [20,21]. Among them, curcumin (Cur) certainly plays a relevant role [22]. Cur is a polyphenolic compound obtained from the dried rhizome (also known as turmeric) of *Curcuma longa* L., an herbaceous perennial plant belonging to the Zingiberaceae family [23]. It is a yellow/orange pigment that is owed the characteristic coloring of curry, a spice particularly used in the culinary field by the Asian population [24]. Thanks to a wide range of pharmacological effects, Cur was extensively used in traditional medicine. Indeed, the ancient Indian medical system, known as Ayurveda, and the traditional Chinese medicine recognized Cur as a remedy for a large variety of diseases, including gastric disorders, hemorrhoids, liver problems, menstrual cramps, wounds (wound repair agent), pregnancy nausea, skin infections or alterations, arthritis, and conjunctivitis [25,26]. In the last decades, a plethora of studies strongly supported its health-benefit effects, highlighting the antioxidant [27], neuroprotective [28,29], anticancer [30], anti-aging [31], antimicrobial [32], anti-inflammatory [28,33], and immunomodulatory [34] properties of Cur in different in vivo and in vitro models. Nevertheless, the therapeutic applications of this natural compound were strongly limited by some drawbacks: low water solubility, chemical instability, poor bioavailability due to insufficient absorption, and rapid metabolism [35]. To increase the bioaccessibility and efficacy, several Cur-loaded nanosystems have been developed [36,37,38,39,40,41,42,43,44,45], which possess higher biological activity due to their subcellular size with respect to non-nanostructured formulations. For instance, nanoencapsulation may enhance the bioactive concentration in food areas, such as water-rich phases or liquid–solid interfaces, where food microorganisms are likely located [46].

Polymeric nanocapsules are nanosystems in which a lipophilic core is surrounded by a polymer wall. Several materials and methods (nanoprecipitation, layer-by-layer deposition, polymer coating, and nanoemulsion template) were used for the preparation of polymer-based nanocapsules. Nanocapsules can serve as drug delivery systems, indeed, they can protect the drug from degradation and improve the bioavailability of the encapsulated drug [47]. Among the utilized polymers to prepare nanocapsules, poly(ε-caprolactone) (PCL) is a biocompatible polyester polymer that has various biomedical applications (absorbable surgical implants and sutures, wound healing, tissue bioengineering) [48]. Furthermore, PCL is biodegradable and fully metabolized in the human body [49,50] but is more stable, and its biodegradation is slower than other polymeric esters, such as polylactide (PLA) and poly(lactic-co-glycolic acid) PLGA [48]. PCL polymer can encapsulate various active ingredients for potential therapeutical applications [51].

Therefore, in the present study, we prepared Cur-loaded nanocapsules (Cur-NCs) based on PCL by interfacial deposition method of a preformed polymer [42,43,44,45,52,53,54,55] and characterized for their physicochemical properties. We observed the stability of Cur-NCs over time and their behavior in simulated gastrointestinal digestion conditions. Moreover, we evaluated the antimicrobial activity of the free and encapsulated Cur against commensal and pathogenic microorganisms, belonging to gut microbiota. Given the tight relationship between infection and inflammation, we also tested the ability of Cur and Cur-NCs to enhance the immunomodulatory activity of ADSCs. To this purpose, we evaluated the capacity of the two Cur forms to modulate the expression levels of pro- and anti-inflammatory cytokines on ADSCs exposed to inflammatory insult, induced by lipopolysaccharide (LPS).

## 2. Results

### 2.1. Characterics of Curcumin Nanocapsules

The Cur nanoencapsulation design consisted of the formation of nanocapsules having a lipophilic core, formed by the dispersant sorbitan monostearate (SM) and the caprylic/capric triglyceride (CCT) in which Cur was allocated. A polymeric wall, constituted by the biodegradable and biocompatible polymer PCL, surrounded the core. The wall is covered by the non-ionic surfactant polysorbate 80 (P80), which conferred hydrophilicity and ensured the stability of the nanocapsule suspension due to steric hindrance of the branched alkyl chain. The method to prepare Cur-NCs [52,53], which leads exclusively to the formation of nanometric-sized capsules, involved the use of an organic phase (OP) able to dissolve PCL, SM, CCT, Cur, and an aqueous phase (AP) containing P80. Once OA was added to the AP, the aqueous nanosuspension of Cur-NCs was obtained by rapid diffusion of the organic solvent into the AP and its following evaporation. The pH of Cur-NCs nanosuspension was 6.1 ± 0.1. Empty nanocapsules (NCs) were prepared similarly but without Cur.

### 2.2. Measurement of the Size and Potential of Curcumin-Nanocapsules

The nanosuspension of Cur-NCs was characterized by dynamic light scattering (DLS) and electrophoretic light scattering (ELS) measurements. Cur-NCs had a mean hydrodynamic diameter (Z-average) of 223 ± 13 nm, which confirmed the nanometric size of nanocapsules. Moreover, a low polydispersity index (PDI) of 0.09 ± 0.03 and narrow particle size distribution indicated excellent homogeneity of the nanoformulation [52,54,55,56,57]. The Zeta potential (ζ) value was −17 ± 3 mV and agreed with the negative charge originating from carboxylic groups present at the polymer extremities.

### 2.3. Determination of Encapsulated Curcumin

The Cur nanosuspension showed an encapsulation efficiency (EE) of 100%, highlighting the validity of the process that leads to the complete encapsulation of Cur. Giacomeli et al. [44] reported the same EE value but for the Cur loading equal to 0.6 mg/mL. In the present work, the quantity of nanoencapsulated Cur is equal to 1 mg/mL with an increase of 40%. By using a protocol that involved a higher amount of the nanocapsule constituents (i.e., more than 3 times for CCT and SM, and more than 1.5 times for PCL and P80), Cur-NCs with EE equal to only 91% were obtained [42]. This makes the Cur-NCs nanosuspension prepared by us more attractive for large-scale preparation both in terms of higher EE (100%) and lower amount of raw material with reduced costs to be sustained.

### 2.4. Determination of the Spectra of Curcumin-Nanocapsules

**Figure 1:**
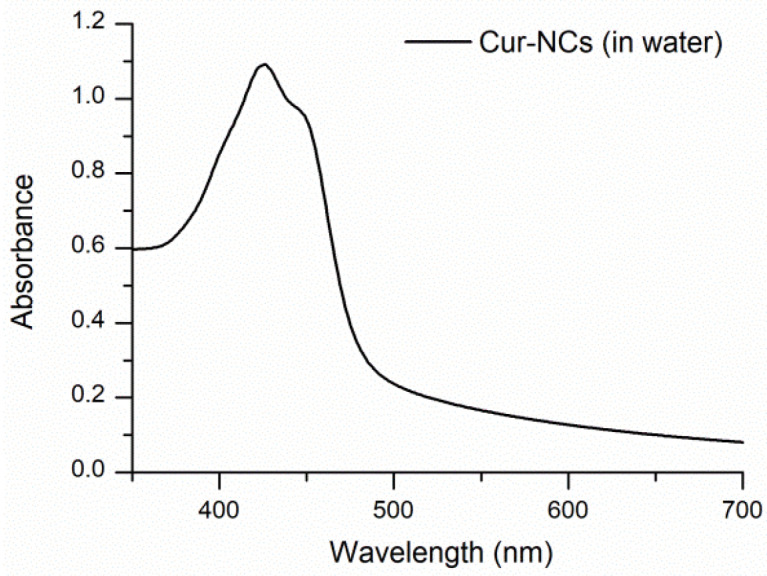
Ultraviolet–visible (UV−Vis) spectrum of curcumin-nanocapsules (Cur-NCs) in water. To perform the UV−Vis spectrum, 10 μL of the Cur-NCs nanosuspension was diluted with 3 mL of pure water.

**Figure 2:**
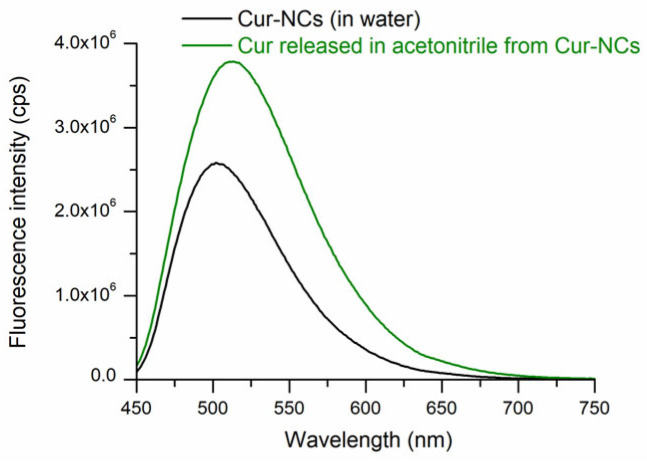
Fluorescence spectra of curcumin-nanocapsules (Cur-NCs) in water and after curcumin (Cur) release in acetonitrile. To perform the fluorescence spectra, 10 μL of the Cur-NCs nanosuspension was diluted with 3 mL of pure water or acetonitrile.

Aqueous yellow nanosuspension of Cur-NCs showed that a typical ultraviolet–visible (UV−Vis) absorption spectrum (λ_max_ = 425 nm) (Figure 1) of Cur in organic solvents, like acetonitrile, methanol, and ethanol, was observed except for the offset baseline due substantially to scattering of NCs. Cur-NCs aqueous nanosuspension showed fluorescent properties in water solvent (Figure 2). When the spectrum was performed in acetonitrile solvent, a higher fluorescence was observed due to the release of the Cur from the nanocapsules and to the solvatochromic effect of the acetonitrile solvent. The maximum wavelength (λ = 499 nm) of the fluorescence spectrum of Cur-NCs (in water) was blue-shifted with respect to that of Cur released (λ = 511 nm) in acetonitrile (Figure 2). This may be ascribable to the more hydrophobic environment constituted by the lipophilic core of the NCs compared to the polar solvent acetonitrile [58]. The fluorescence properties of Cur-NCs could make them attractive for biological applications.

### 2.5. Effect of the Time on Curcumin-Nanocapsules

**Figure 3:**
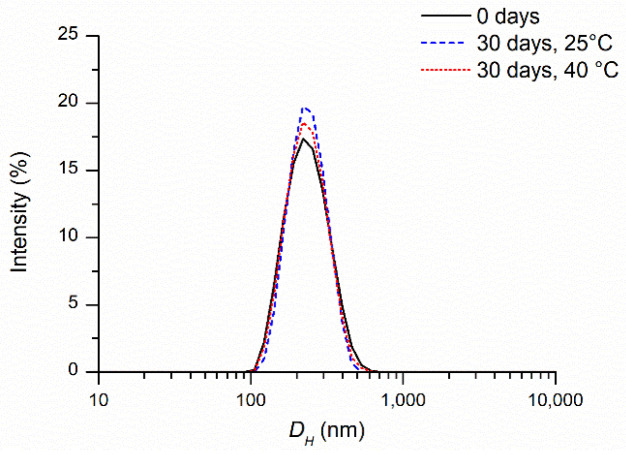
Intensity-weighted distribution of the hydrodynamic diameter (*D_H_*) of freshly prepared curcumin-nanocapsules, after 30 days of storage at 25 °C and 40 °C.

**Figure 4:**
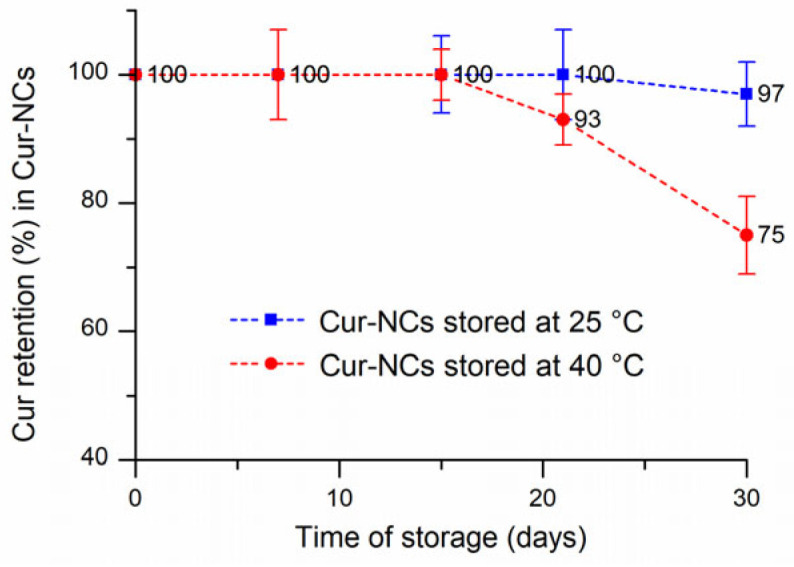
Curcumin (Cur) retention percentage in curcumin-nanocapsules (Cur-NCs) after 30 days of storage at 25 °C and 40 °C.

The results reported in Table 1 and Table 2 and Figure 3 indicated that over 30 days the particle size of the Cur-NCs did not undergo noteworthy changes when stored at 25 °C and 40 °C to simulate thermal stress. The PDI was lower than 0.1 in all experiments. These values agreed with almost monodisperse samples [56] and confirmed the high stability and homogeneity of the Cur-NCs nanosuspension. The ζ value remained negative and underwent little changes during storage (Table 2 and Table 3). The realized system, therefore, was stable and no flocculation or coalescence phenomena were observed during this storage period either at a temperature of 25 °C or at 40 °C. For the sample held at 25 °C, the amount of encapsulated Cur negligibly changed over the time storage, indicating high retention and protection from degradation thanks to the nanoencapsulation process (Table 1, Figure 4). Even in conditions of thermal stress (40 °C), the encapsulated Cur amount was significantly unchanged for up to 21 days. A decrease of 25% was observable after 30 days of storage at 40 °C. Finally, a Cur-NC sample stored for 2 years at 25 °C showed a good Cur retention (81%) confirming the noteworthy stability of Cur-NCs.

### 2.6. Determination of the Stability and Bioaccessibility of Curcumin-Nanocapsules

Simulated digestion tests are based on rapid, cost-effective, and ethically unrestricted methods. They simulate in vivo physiological gastrointestinal conditions and are useful for assessing the stability and absorption of pharmaceuticals and food products in these biological compartments.

Cur-NCs were stable after simulated gastric digestion, as shown in Table 3. The particle size and the PDI did not undergo noteworthy variations, and no aggregation and/or flocculation phenomena were observed. The ζ value of −3 ± 1 mV was attributable to the strongly acidic environment that determines the free carboxyl groups protonation of PCL polymer, resulting in a lower number of negative charges on the nanocapsule surface. To confirm this, after the addition of bicarbonate to the digest up to pH 7, the ζ value reached −24 ± 1 mV (high degree of deprotonation of the polymer). Furthermore, the retention percentage of Cur quantified by high-performance liquid chromatography (HPLC) was noteworthy and amounted to 91 ± 5%.

After simulated intestinal digestion, the Cur stability (S) value was 89 ± 13%. This result indicated that a significant quantity of Cur (not degraded) was present in the intestinal digestion phase. Micellar phase analyses were carried out to determine the Cur bioaccessibility (B) value. The value observed for Cur-NCs was high (96 ± 15%). Finally, the value of the effective bioaccessibility (EB) of nano-encapsulated Cur was equal to 86 ± 13% (for calculation of S, B, and EB, see Equations (3)–(5) in Section 4.5.3 of Materials and Methods) [41,55,59].

### 2.7. Antibacterial Activity of Curcumin-Nanocapsules

The antibacterial effect of NCs, Cur, Cur-NCs, and gentamycin (Gen) was determined through the microdilution method [60] against eight different American Type Culture Collection (ATCC) strains. The results are shown in Table 4. NCs (negative control) were inactive against all the tested ATCC strains. Cur was able to inhibit the growth of *Staphylococcus aureus* (*S. aureus*) ATCC 6538 (MIC 62.50 µg/mL), *Enterococcus faecalis* (*E. faecalis*) ATCC 29212 (MIC 7.81 µg/mL), *Lactobacillus delbrueckii* (*L. delbrueckii*) ATCC 11842 (MIC 250.00 µg/mL), *Escherichia coli* (*E. coli*) ATCC 8728 (MIC 62.50 µg/mL), and *Pseudomonas aeruginosa* (*P. aeruginosa*) ATCC 9027 (MIC 250.00 µg/mL). When Cur was loaded in NCs (Cur-NCs), a loss of activity against Gram-positive bacteria and an improvement of the effect against the Gram-negative strain *E. coli* ATCC 8728 were observed. In the latter case, a reduction in one dilution factor (MIC 31.25 µg/mL) was shown compared to unencapsulated Gen (MIC 62.50 µg/mL). The Clinical and Laboratory Standards Institute (CLSI) interpretive criteria were applied to establish the susceptibility of bacteria against the standard antibiotic Gen [61,62]. Specifically, three out of four *Lactobacillus* strains showed resistance to Gen. The Gram-positive strain *S. aureus* and the Gram-negative bacteria *E. coli* and *P. aeruginosa* were susceptible to the standard antibiotic. Regarding *E. faecalis* strain, no interpretive criteria for Gen are reported in CLSI M100-S30 [61].

### 2.8. Effect of Free and Encapsulated Curcumin on Human Adipose-Derived Stem Cells Viability

**Figure 5:**
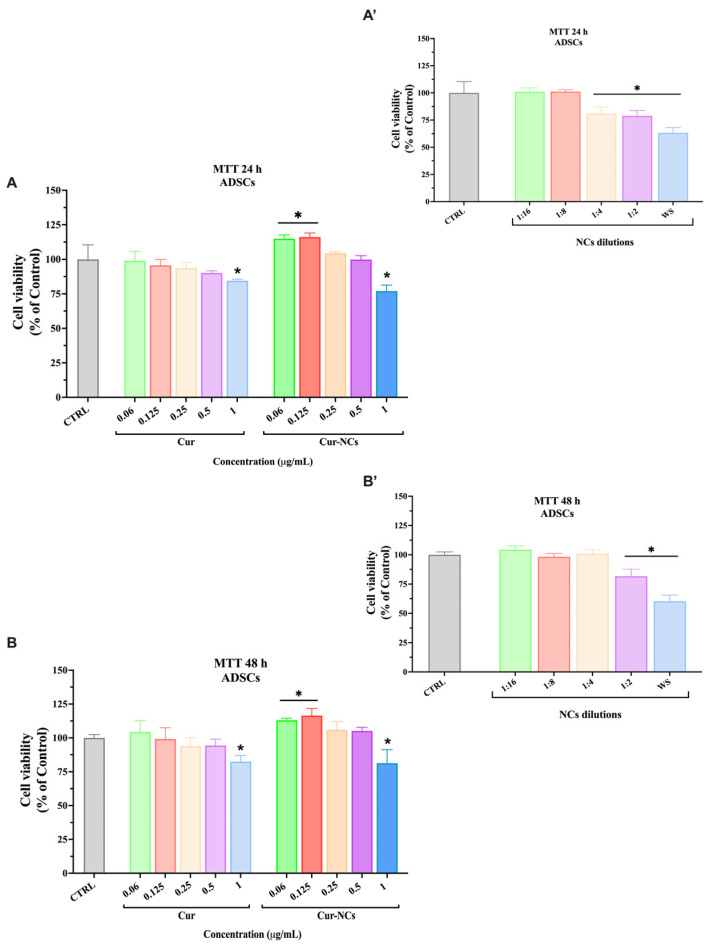
Dose and time-dependent effects of curcumin (Cur), empty nanocapsules (NCs), and curcumin-loaded nanocapsules (Cur-NCs) on primary human adipose-derived stem cells (ADSCs) viability. Cells were grown in a culture medium (CTRL) or exposed to increasing concentrations (0.06–1 µg/mL) of Cur or Cur-NCs or different dilutions of NCs for 24 h (**A**,**A’**) and 48 h (**B**,**B’**). Results are expressed as a percent of control. The bars represent means ± SD from three independent experiments performed in triplicate (SD = standard deviation). Statistically significant differences, determined by one-way analysis of variance ANOVA and the Tukey post-test, are indicated: * *p* < 0.05 vs. CTRL at the same incubation time. WS: working standard: diluted solution derived from the stock solution.

The possible cytotoxic effect exerted by Cur, NCs, and Cur-NCs on primary human ADSCs was evaluated by the 3-[4,5-dimethylthiazol-2-yl]-2,5-diphenyl tetrazolium bromide (MTT) assay. Therefore, ADSCs were treated with increasing concentrations (0.06–1 µg/mL) of Cur and Cur-NCs for 24 h and 48 h (Figure 5A,B). To evaluate the cytotoxicity of the vehicle, ADSCs were treated with NCs, diluted in the same ratio used for Cur and Cur-NCs, and incubated for 24 h and 48 h (Figure 5A’,B’).

The treatment of ADSCs with the highest tested concentration of Cur induced a significant reduction in cell viability, at both time points (Figure 5A,B). Concerning the vehicle, at 24 h, the treatment with the NCs caused a significant reduction in ADSCs viability already at the 1:4 dilution ratio (Figure 5A’), whereas, at 48 h, a similar reduction was observed at the 1:2 dilution ratio (Figure 5B’). Such dilution ratios correspond to the dilution used to obtain the concentration of 0.25 µg/mL and 0.5 µg/mL of Cur and Cur-NCs, respectively. However, the cytotoxic effect of the NCs was abolished when they were loaded with Cur. Indeed, the treatment of ADSCs with 0.06 and 0.125 µg/mL Cur-NCs induced a significant increment of cell viability, at both time points. No variation of cell viability was observed in ADSCs treated with 0.25 and 0.5 µg/mL Cur-NCs, at 24 h as well as 48 h. Similar to the free form, only the highest tested concentration (1 µg/mL) of Cur-NCs was able to affect ADSCs viability, at both 24 h and 48 h (Figure 5A,B). Based on these results, we chose 0.125 μg/mL as the concentration for ADSCs treatment in the subsequent experiments.

### 2.9. Effect of Curcumin and Curcumin-Nanocapsules on Human Adipose-Derived Stem Cells Viability in an Inflammatory Context

**Figure 6:**
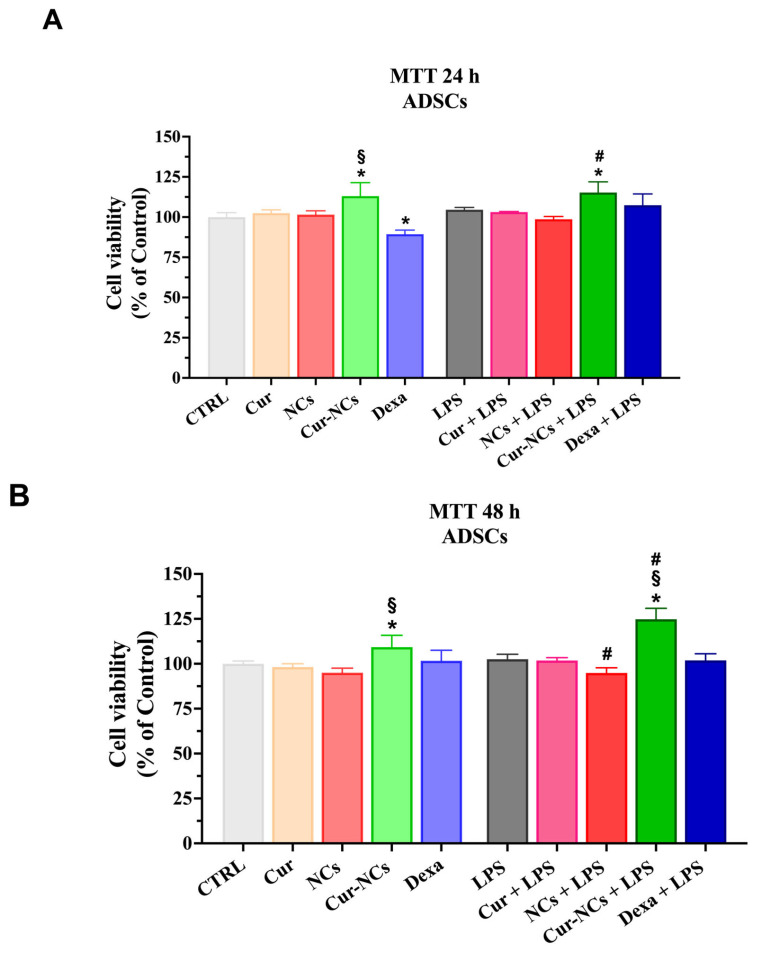
Effect of curcumin (Cur), empty nanocapsules (NCs), curcumin-loaded nanocapsules (Cur-NCs), and dexamethasone (Dexa) on primary human adipose-derived stem cells (ADSCs) cell viability, at the steady state, and under inflammatory stimuli. Cells were grown alone (control: CTRL) or in the presence of 0.125 µg/mL of Cur or Cur-NCs, NCs diluted in the 1:8 ratio, 10 nM Dexa, with or without 1 µg/mL lipopolysaccharide (LPS), for 24 h and 48 h. Histograms showed ADSCs cell viability, at 24 h (**A**) and 48 h (**B**). Results are expressed as a percent of control. The bars represent means ± SD from three independent experiments performed in triplicate (SD = standard deviation). Statistically significant differences, determined by one-way analysis of variance ANOVA and the Tukey post-test, are indicated: * *p* < 0.05 vs. CTRL; § *p* < 0.05 vs. Cur; # *p* < 0.05 vs. LPS, at the same incubation time.

To verify a possible anti-inflammatory action of Cur-NCs, we introduced, in our experimental design, the treatment with LPS, an outer membrane component of Gram-negative bacteria, able to induce an inflammatory state. Furthermore, dexamethasone (Dexa) was used as a standard agent to compare the potential anti-inflammatory activity of the Cur-NCs. Accordingly, we analyzed ADSCs cell viability in our experimental conditions by MTT assay. Therefore, ADSCs were grown in a normal culture medium (Control; CTRL) or the presence of 0.125 µg/mL of Cur or Cur-NCs, NCs diluted in the 1:8 ratio, 10 nM of Dexa with or without 1 µg/mL of LPS, for 24 h and 48 h (Figure 6A,B). NCs 1:8 dilution ratio corresponds to the dilution used to obtain the concentration of 0.125 µg/mL of Cur and Cur-NCs.

At 24 h, the treatment of ADSCs with Cur-NCs determined a significant increment of cell viability compared to the Control and Cur. Moreover, the treatment of ADSCs with Dexa induced a significant reduction in cell viability compared to the Control. Interestingly, the exposition of ADSCs to LPS did not produce any variation in cell viability. Noteworthy, a significant increase in cell viability was observed in ADSCs concomitantly treated with Cur-NCs and LPS, compared to both unstimulated (Control) and LPS-stimulated cells (Figure 6A).

At 48 h, a significant increment in cell viability was observed when ASCs were exposed to Cur-NCs compared to both Control and Cur. Once again the treatment of ADSCs with LPS did not cause any change in cell viability, indicating the high resistance of these cells to inflammatory insults. The simultaneous treatment of ADSCs with NCs and LPS caused a slight but significant reduction in cell viability compared to LPS-stimulated cells. Interestingly, a significant increase in cell viability was observed in ADSCs concomitantly treated with Cur-NCs and LPS compared to unstimulated (Control) and LPS-stimulated cells (Figure 6B).

### 2.10. Evaluation of Apoptotic Cell Death on Human Adipose-Derived Stem Cells Exposed to Curcumin or Curcumin-Nanocapsules in the Presence or Absence of Inflammatory Stimuli

**Figure 7:**
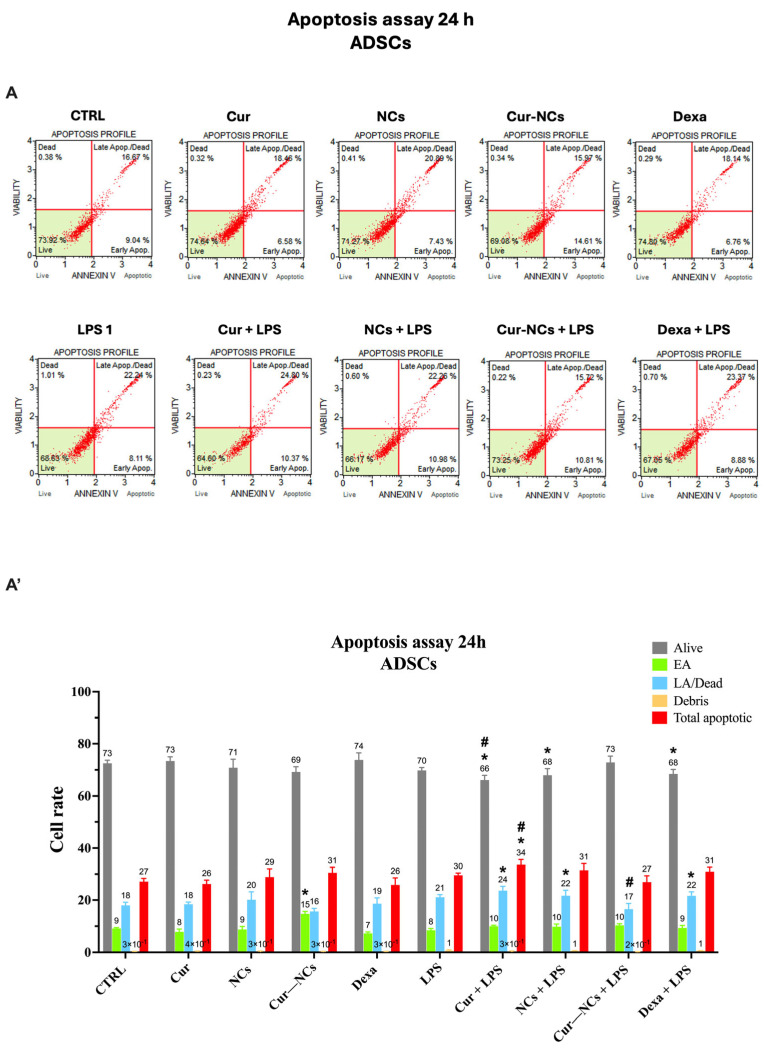

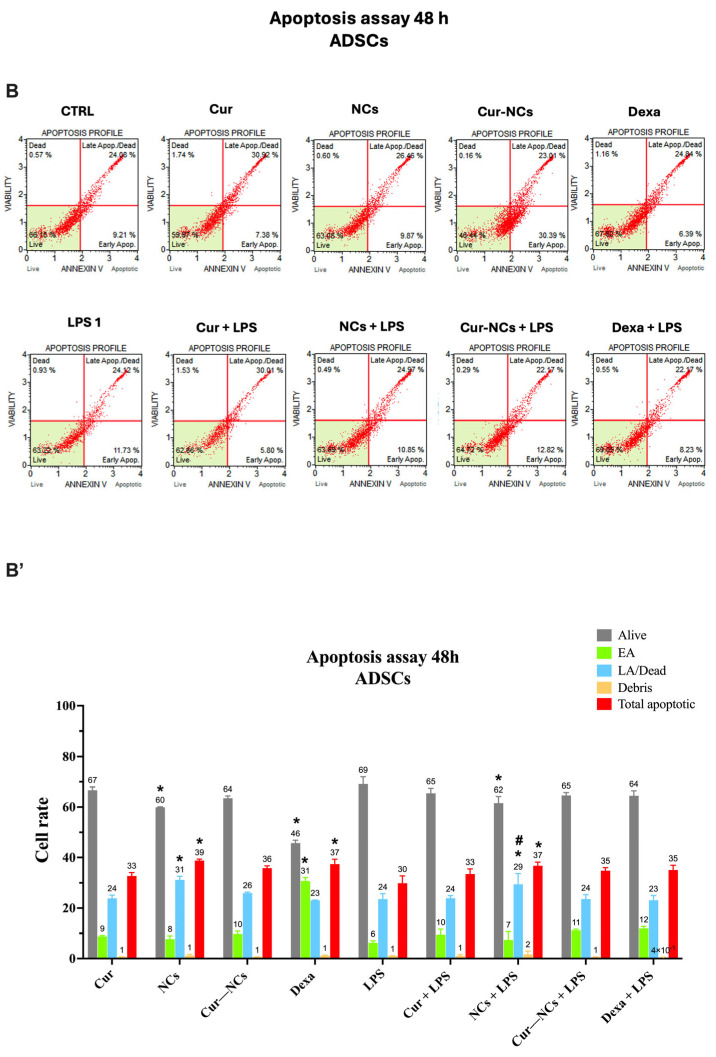
Evaluation of apoptotic cell death on human adipose-derived stem cells (ADSCs) exposed to curcumin (Cur), empty nanocapsules (NCs), curcumin-loaded nanocapsules (Cur-NCs), dexamethasone (Dexa), and lipopolysaccharide (LPS) in the presence or absence of inflammatory stimuli. A–B Scatter plots of ADSCs grown, for 24 h (**A**) and 48 h (**B**), in normal culture medium (control; CTRL) or the presence of 0.125 µg/mL Cur or Cur-NCs, NCs diluted in the 1:8 ratio, 10 nM Dexa, with or without 1 µg/mL LPS. Each plot reports four squares in which cells are distributed based on their staining. (**A’**,**B’**) Histograms showed the rate of vital cells (Alive), early apoptotic cells (EA), late apoptotic (LA)/dead cells, and debris for each experimental condition, at 24 h (**A’**) and 48 h (**B’**). The bars represent means ± SD of three independent experiments (SD = standard deviation). Statistically significant differences, determined by two-way analysis of variance ANOVA and the Tukey post-test, are indicated: * *p* < 0.05 vs. CTRL; **#** *p* < 0.05 vs. LPS, at the same incubation time.

To further explore the behavior of ADSCs in our experimental conditions, we evaluated the apoptotic cell death by Muse^®^ cell Analyzer. Cells were cultured in a normal culture medium (Control; CTRL) or the presence of 0.125 µg/mL of Cur or Cur-NCs, NCs diluted in the 1:8 ratio, 10 nM of Dexa with or without 1 µg/mL of LPS, for 24 h (Figure 7A,A’) and 48 h (Figure 7B,B’). The scatter plots shown in Figure 7A,B represent the distribution of the cells in four squares, based on their staining. Accordingly, it is possible to distinguish four cell populations: viable cells (Alive), early apoptotic cells (EA), late apoptotic (LA) cells/dead cells, and cell debris. The total apoptotic cells represent a further group that includes the EA and the LA/dead cell populations.

At 24 h, in the absence of LPS stimulation, a significant increment of the EA cell rate in ADSCs treated with Cur-NCs, compared to the untreated cells (Control) and Cur, was observed. According to MTT results, the stimulation of ADSCs with LPS did not induce any variation in cell rates compared to unstimulated cells (control). Furthermore, the simultaneous addition of LPS to Cur, NCs, and Dexa provoked a significative reduction in vital cells and a concomitant increase in the LA/dead cells with respect to control. Such increment reflects the significant increase in the total apoptotic cell rates observed in ADSCs concomitantly treated with Cur and LPS compared to unstimulated and LPS-stimulated cells. Interestingly, the harmful effect of LPS on ADSCs was counteracted by the treatment with Cur-NCs. Indeed, in this case, the rate of alive and EA cells was similar to the control, whereas the LA/dead cell rates were significantly reduced compared to that observed on LPS-stimulated ADSCs (Figure 7A,A’).

At 48 h, a significant reduction in vital cell rate and a contextual increase in LA/dead cell rate were revealed on ADSCs treated with 0.125 µg/mL Cur, with or without LPS, compared to the control. Consistently, the total apoptotic cell population was significantly higher in ADSCs treated with 0.125 µg/mL Cur, with or without LPS, compared to the control. Furthermore, in the absence of inflammatory stimuli, the treatment of ADSCs with Cur-NCs caused a significant reduction in vital cell rate, followed by a concomitant increase in EA cell rate compared to the control. Such increment is responsible for the significant enhancement in the total apoptotic cell population in ADSCs treated with Cur-NCs compared to the steady state (control). However, early apoptosis is a reversible cellular event that can be reverted to healthy conditions. Interestingly, this phenomenon did not occur in the presence of inflammatory stimuli. Indeed, the addition of LPS to Cur-NCs did not induce any variation in the rates of all cell populations compared to the control.

### 2.11. Immunomodulatory Effect of Curcumin-Nanocapsules on Human Adipose-Derived Stem Cells Grown in the Presence or Absence of Inflammatory Stimuli

**Figure 8:**
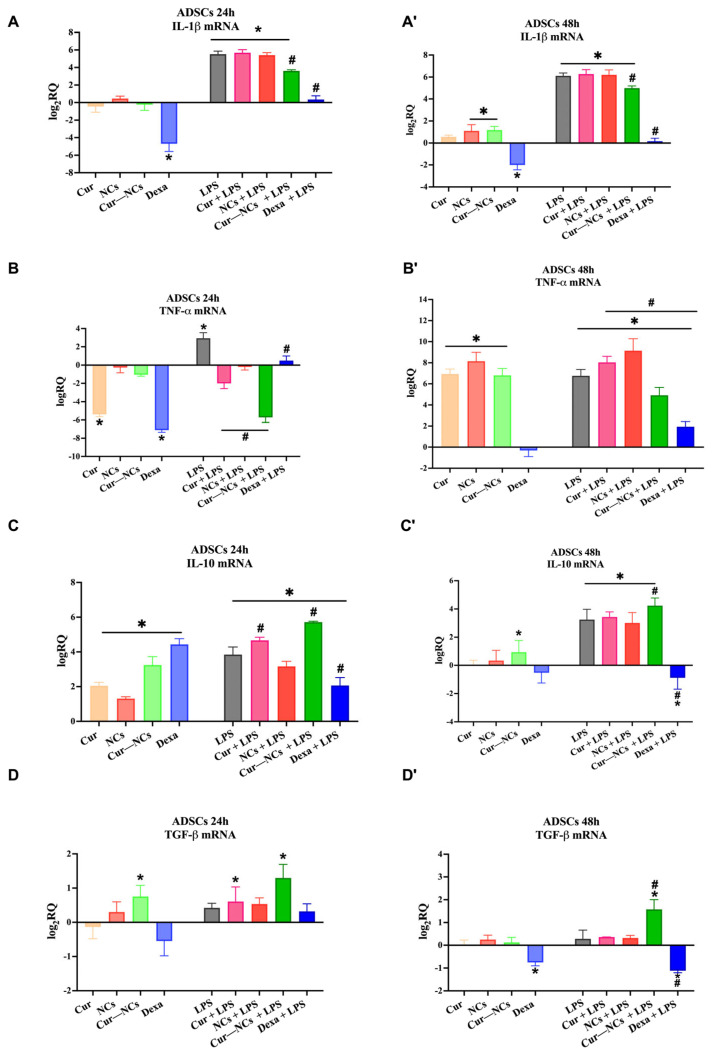
Expression levels of inflammatory cytokines IL-1β, TNF-α, IL-10, and TGF-β on human adipose-derived stem cells (ADSCs) exposed to curcumin (Cur), empty nanocapsules (NCs), curcumin-loaded nanocapsules (Cur-NCs), dexamethasone (Dexa), with or without lipopolysaccharide (LPS), for 24 h and 48 h. ADSCs were cultured in normal culture medium (control; CTRL) or the presence of 0.125 µg/mL Cur or Cur-NCs, NCs diluted in the 1:8 ratio, 10 nM Dexa, with or without 1 µg/mL LPS, for 24 h (**A**–**D**) and 48 h (**A’**–**D’**). Histograms showed IL-1β, TNF-α, IL-10, and TGF-β mRNA expression levels on ADSCs grown in our experimental conditions for 24 h (**A**–**D**) and 48 h (**A’**–**D’**). The bars represent means ± SD of three independent experiments (SD = standard deviation). Statistically significant differences, determined by one-way analysis of variance ANOVA and the Tukey post-test, are indicated: * *p* < 0.05 vs. CTRL; # *p* < 0.05 vs. LPS, at the same incubation time.

To verify the possible immunomodulatory action of Cur-NCs, we tested the ability of this Cur-delivery system to modulate the expression of both pro- and anti-inflammatory cytokines on ADSCs at the steady state or stimulated with LPS. Figure 8 shows the mRNA expression levels of two pro-inflammatory cytokines, including IL-1β and TNF-α, and two anti-inflammatory cytokines, such as IL-10 and TGF-β on human stem cells grown in a normal culture medium (Control; CTRL) or the presence of 0.125 µg/mL of Cur or Cur-NCs, NCs diluted in the 1:8 ratio, 10 nM of Dexa with or without 1 µg/mL of LPS, for 24 h (Figure 8A,C) and 48 h (Figure 8A’,D’).

Concerning the IL-1β, at 24 h, in the absence of inflammatory stimuli, the mRNA expression level did not vary compared to the control, except in the presence of Dexa, which induced a significative reduction in IL-1β expression with respect to the control. As expected, the treatment of ADSCs with LPS alone or in combination with Cur, NCs, and Cur-NCs significantly upregulated IL-1β mRNA expression. Interestingly, cotreatment of Cur-NCs and Dexa with LPS caused a significant reduction in the IL-1β expression levels compared to the LPS alone (Figure 8A). A similar trend was found at 48 h (Figure 8A’). However, at this time point, in the absence of inflammatory insult, the exposition of ADSCs to the NCs or Cur-NCs caused a slight but significant increase in IL-1β transcription level (Figure 8A’).

As mentioned, we also investigated the expression level of a further pro-inflammatory cytokine: TNF-α. In this case, at 24 h, in the absence of LPS, the treatment with Cur and Dexa significantly reduced the expression level of the cytokine compared to the control. The addition of LPS to the culture medium induced a significant increment in the TNF-α mRNA levels, suggesting the activation of inflammatory response. The simultaneous addition of LPS to Cur, NCs, Cur-NCs, and Dexa caused a significant reduction in TNF-α expression levels, particularly in the presence of Cur-NCs (Figure 8B). Conversely, at 48 h, in the absence of LPS, TNF-α was significantly overexpressed compared to the control, in all experimental conditions, except for Dexa, which did not induce any variation in the cytokine expression. In the presence of LPS, the treatment of ADSCs with Cur or NCs induced a significant increment in TNF expression compared to the LPS alone. Noteworthy, the treatment of stem cells with LPS and Cur-NCs or Dexa determined a significant reduction in TNF-α expression level compared to LPS alone (Figure 8B’).

The expression analysis of IL-10 on ADSCs, exposed, for 24 h, to our experimental conditions revealed a significant upregulation of this cytokine. The addition of Cur or Cur-NCs, alone or in combination with LPS, determined a significant enhancement of the IL-10 expression compared to the control and LPS. However, this effect was more evident for Cur-NCs. In the presence of LPS, the standard drug Dexa significantly reduced the mRNA level of the cytokine compared to the LPS alone, suggesting a repressor action of Dexa against both pro- and anti-inflammatory cytokines (Figure 8C). At 48 h, in the absence of inflammatory stimuli, the treatment of ADSCs with Cur, Cur-NCs, or Dexa did not determine any change in IL-10 mRNA expression with respect to the control. On the other hand, the exposition of ADSCs to Cur-NCs induced a small but significant increment in cytokine expression. The addition of LPS to the normal culture medium or the other treatments produced a significant increment in the IL-10 mRNA levels compared to the control. Interestingly, in the presence of inflammatory stimuli, the treatment of ADSCs with Cur-NCs caused a significant increment in IL-10 expression compared to the LPS. The simultaneous addition of Dexa to LPS significantly downregulated the IL-10 mRNA expression (Figure 8C’).

Regarding the TGF-β, a significant increase in the expression of the anti-inflammatory cytokine in ADSCs treated for 24 h with Cur-NCs, alone or in combination with LPS, compared to the control was observed (Figure 8D). A similar increment of TGF-β mRNA levels was found in human stem cells concomitantly treated with Cur and LPS, indicating that Cur, in both free and encapsulated form, plays a protective role in an inflammatory context. Conversely, at 48 h, the treatment of ADSCs with Dexa induced a significant reduction in TGF-β expression, mainly in the presence of LPS. It is worth noting that in the presence of LPS, the exposition of ADSCs to Cur-NCs determined a significative increment in cytokine expression (Figure 8D’).

## 3. Discussion

Chronic inflammation is a key driver of numerous degenerative diseases, including cancer, diabetes, and cardiovascular disorders [7]. Nutraceuticals with anti-inflammatory properties, such as Cur, have been extensively studied for their potential therapeutic benefits. However, Cur’s clinical application has been limited by its poor bioavailability. To address this, PCL nanocapsules were developed, offering a promising strategy to enhance Cur’s bioavailability and therapeutic efficacy. In this study, Cur-NCs demonstrated significant potential in improving Cur’s stability, bioaccessibility, and immunomodulatory activity. These findings suggest that Cur-NCs could be an effective therapeutic option for treating inflammation-related diseases and maintaining gut health [63]. However, the beneficial effects of Cur on human health are strongly limited by its poor water solubility and scarce bioavailability, due to a rapid metabolism at the gastrointestinal level [64]. To overcome these obstacles, different nanotechnologies were developed [65,66]. Nanoencapsulation is a technique that allows to protect the bioactive compounds from adverse conditions, increasing solubility and physical stability. The nanoscale size is characterized by physicochemical properties such as high surface-to-volume ratio and particular reactivity with biological systems. Recently, polymer-based nanocapsules have been gaining increasing interest in medical applications. PCL is a biodegradable and biocompatible polymer, which is completely metabolized into non-toxic byproducts, such as 6-hydroxycaproic acid, in the human body [49]. Furthermore, compared to other biodegradable polymers, like PLA and PLGA, PCL degrades at a slower rate. This slow biodegradation is advantageous for applications requiring prolonged material presence, such as drug delivery systems and long-term implants [67].

Accordingly, in the present study, Cur-NCs, synthesized by the nanoprecipitation method, showed high homogeneity of particle population (PDI < 0.1) and EE (100%). Moreover, the fluorescence features of Cur-NC formulation could be useful for biomedical applications (such as diagnostic or theranostic agents). The Cur-NCs remained stable at both 25 °C and 40 °C (thermal stress conditions), exhibiting high particle size stability and Cur retention.

Therefore, the NCs could be useful reservoirs and slow-release systems for Cur in contact with biological compartments. We also verified the noteworthy stability of a simulated gastric environment. This highlights that the nanocapsules effectively protected Cur during gastric digestion and that only a small loss of active ingredient was detectable. After an experiment simulating intestinal digestion, Cur-NCs provided Cur dispersed in the micellar phase, capable of being absorbed by the intestine. A high B value of 96% was calculated. The comparison with other delivery systems loading Cur such as liposomes or micelles [68] has highlighted the better stability and bioaccessibility of Cur-NCs. For example, Cheng et al. [69] reported that the amount of encapsulated Cur in liposome by thin film method, stored for 1 month at 4 °C, decreased from 78.6% to 50.1% and bioaccessibility was 55.4%. Our findings encourage the potential oral administration of Cur-NCs in biomedical devices or fortified foods. The development of fortified food can be considered a good strategy to improve the intake of Cur, thus promoting the health-benefit effect of this natural compound at both local and systemic levels.

Focusing on the gastrointestinal tract, a clinical study proved the ability of Cur to reduce digestive complaints, without affecting the intestinal microbiota [70]. Gut microbiota consists of a heterogeneous group of microorganisms, including bacteria, archaea, eukarya, viruses, and parasites [71]. All these organisms are essential for the host‘s physiological functions [72]. Indeed, they are involved in immune system homeostasis, regulation of host metabolism, prevention of pathogens invasion, and improvement of the epithelial barrier function [73,74]. However, by sharing the same niche, microorganisms interact with each other and the host. Such interaction is characterized by a delicate equilibrium, and alterations in the microbiota composition can seriously compromise the host’s health.

Among the gut-colonizing microorganisms, probiotics represent live microorganisms that, when administered in adequate amounts, contribute to the hosts’ health and prevent several diseases [75]. An example of probiotic bacteria is the members of the genus *Lactobacillus* (phyla Firmicutes), which play a protective role, ensuring the gut barrier integrity and mucosal barrier defense and promoting the host immune responses [73]. In this regard, natural bioactive compounds can help create an ideal environment for the growth of probiotic bacteria, including *Lactobacillus* species, and they are useful in treating gastrointestinal dysbiosis and inflammation [76]. Some natural compounds can act as prebiotics, namely, dietary substances that are not digested by humans but are selectively utilized by gut bacteria for their growth [77]. In our study, Cur-NCs did not inhibit the growth of *Lactobacillus* spp., corroborating the role of this compound as a prebiotic. Indeed, recent studies have highlighted the positive effects of Cur on probiotic bacteria [78,79,80]. These effects are achieved thanks to the decrease in LPS production and the increase in intestinal barrier permeability [78], the inhibition of the growth of Gram-positive and Gram-negative pathogenic bacteria [79], and the protection of beneficial bacteria, including *Lactobacilli* and *Bifidobacteria* [80]. Cur’s beneficial effect on the growth of Lactobacilli was explained by the capacity of this polyphenol to improve the uptake of different nutrients and act as a substrate for bacteria [81]. In an in vivo study in a rat model, Xu et al. demonstrated the protective effect of Cur on gut microbiota [82]. Specifically, Cur reduced the growth of opportunistic pathogens strains, like *E. coli*, and increased the concentration of short-chain fatty acids (SCFAs) producers bacteria, such as *Lactobacillus* spp. [82]. It was demonstrated that SCFAs exerted a protective function on the gut, improving barrier function and reducing intestinal inflammation [83].

Together with probiotic bacteria, *E. coli* represents one of the most common Gram-negative bacteria found in the intestinal tract of humans and other warm-blooded animals [84,85]. This bacterium colonizes the human gut after childbirth, and as commensal, it lives in a beneficial association with the host [86,87]. *E. coli* can produce several substances useful to the host, such as vitamin K and B_12_ [88]. As an anaerobe facultative bacterium, it depletes oxygen diffusing from gut mucosa, creating a favorable environment for strict anaerobe bacteria growth. Anaerobic bacteria break down complex polysaccharides in the gut, producing simple carbohydrates essential for *E. coli* growth [89,90]. However, alteration of the intestinal barrier and immune dysfunction in the host can determine the switch of *E. coli* from a commensal microorganism to an opportunistic pathogen [91]. Pathogenic *E. coli* strains are responsible for several diseases, including gastrointestinal [92] and urinary [93] tract infections, pneumonia [94], meningitis [95], and sepsis [96]. This is due to the capacity of the microorganism to modify its genome architecture and persist in the host’s environment, colonizing different niches. In effect, it is well known that *E. coli* can acquire specific virulence factors and antimicrobial resistance traits through plasmids, bacteriophages, and transposons [97].

In our work, Cur-NCs efficiently inhibited *E. coli* growth at 31.25 µg/mL, reducing the MIC of a dilution factor with respect to Cur (MIC 62.50 µg/mL). Our results highlight the efficacy of Cur-NCs against pathogenic bacteria and are supported by the work of Kapustová et al., in which biocompatible PCL nanocapsules loaded with essential oils (EOs) from *Thymus capitatus* and *Origanum vulgare* inhibited the growth and biofilm formation of *E. coli* and *S. aureus* strains. Furthermore, this nanosystem exhibited higher antifungal activity against *Candida albicans* with respect to the free form of EOs [98]. The antibacterial effect of Cur against *E. coli* may be attributed to different molecular and cellular mechanisms. Adeyemi et al. demonstrated that Cur can induce oxidative stress in *E. coli*, leading to reactive oxygen species (ROS) generation and DNA fragmentation [99]. The inhibition of *E. coli* growth was also attributed to Cur induction of cell wall permeabilization [100] and its interaction with the active site of FtsZ, a cytoskeletal protein involved in prokaryotic cell division [101]. Finally, Cur can interfere with *E. coli* quorum sensing signal transmission, preventing the development of bacterial biofilm and microcolonies [102].

In the gut, the production of biologically active substances by the resident microbiota modulates the delicate balance between anti-inflammatory and pro-inflammatory responses [76]. Specifically, inflammation represents a primary response to external insults. Among them, bacterial invasion is certainly one of the most common inductors.

In recent decades, growing interest has focused on the protective role of mesenchymal stem cells (MSCs). In particular, ADSCs, a cell population of the vascular stroma of adipose tissues, are endowed with self-renewing and multi-differentiation potential. Compared to other stem cell sources, such as bone marrow, ADSCs can be obtained with less invasiveness and higher yield, further enhancing their clinical utility [103]. Moreover, ADSCs show scarce immunogenicity, due to the low or absent expression of immunogenic surface antigens (CD40, CD40L, CD80, and CD86) and major histocompatibility complex I and II [104,105]. All these characteristics make ADSCs eligible for allogeneic cell-based therapies [106,107,108].

It is worth highlighting that ADSCs proved in vitro immunomodulatory activity. It has been shown that ADSCs are able to reduce the inflammatory/immune response, by inducing the polarization of macrophages toward anti-inflammatory M2 macrophages. Such a process is mediated by the release of various soluble molecules, including the TGF-β [109]. Furthermore, ADSCs exerted their immunomodulatory activity by producing anti-inflammatory cytokines such as IL-10 and Arginase-1 and reducing the production of proinflammatory cytokines like TNF-α, IL-12, and IL-1β [17,18,110].

In light of these considerations, in the present study, we proposed to investigate whether Cur-NCs are capable of potentiating the immunomodulatory activity of LPS-stimulated ADSCs. To this purpose, we first analyzed the possible toxic effect of the free (Cur) and encapsulated (Cur-NCs) forms of Cur and the vehicle (NCs) on ADSCs. The results revealed the absence of the cytotoxic effect of both forms of Cur, except at the highest tested concentration (1 μg/mL). In fact, at this concentration, Cur, in both forms, induced a slight but significant decrease in ADSC cell viability at both 24 h and 48 h. Our findings are supported by Kunwar et al. who reported a cytotoxic effect of Cur on both tumoral (EL4, murine T-cell lymphoma cells and MCF7, human breast cancer cells) and normal (NIH3T3, mouse fibroblast cells) cell lines, although cancer cells were more susceptible to Cur treatment compared to the normal ones [111]. A further study proved a dose-dependent cytotoxic effect of Cur on rat peripheral blood lymphocytes. The authors attributed this harmful effect to an increased lipid peroxidation induced by Cur. However, under oxidative stress, Cur promoted the activation of the antioxidant enzymes, thus showing a ROS scavenger effect [112]. On the other hand, the treatment of ADSCs with the lower tested concentration (0.06 and 0.125 μg/mL) of Cur-NCs determined a significant increment in ADSCs cell viability at both time points.

Our results suggest that the NCs can vehicle Cur inside the cells, thus allowing Cur to exert its protective action. Our hypothesis is supported by Pohlmann et al. who reported that PCL is a very efficient drug delivery system since it can modulate the release of drugs, enhance their solubility in aqueous media, and improve photochemical stability [51].

However, a dose-dependent reduction in ADSC cell viability following the treatment with NCs was observed, indicating the ability of the vehicle to interfere with ADSC metabolic activity. Such a harmful effect was abolished when NCs were loaded with Cur, thus highlighting the positive effect of Cur-NCs on ADSC cell viability. In our previous paper, we found a similar effect of NCs on the human keratinocyte cell line (HaCaT) [98]. Nevertheless, the genotoxic analysis did not reveal any DNA damage in HaCaT cells treated with NCs, compared with untreated control cells. Therefore, although NCs affect ADSC cell viability, it is reasonable to hypothesize that they did not cause severe cell damage [98]. Based on cell viability results, we selected the subtoxic concentration of 0.125 μg/mL for the subsequent experiments.

To evaluate the ability of Cur-NCs to enhance the immunomodulatory activity of ADSCs, we exposed stem cells to pro-inflammatory stimuli, induced by LPS. This molecule is normally present on the outer membrane (OM) of Gram-negative bacteria such as *E. coli* [113]. As a component of OM, LPS ensures structural integrity and reduces membrane permeability, thus preventing the passage of damaging molecules, such as antibiotics and cationic antimicrobial peptides [114]. For this reason, Gram-negative bacterial infections are generally difficult to treat. However, LPS exerts an immunogenic action, since it is recognized by PRRs (e.g., Toll-like receptor-4, TLR-4), expressed by innate immune cells [115]. TLR-4-LPS interaction triggers a signaling cascade that culminates in the activation of NF-κB, a transcription factor involved in inflammatory cytokines synthesis [116,117]. In our case, the exposition of ADSCs to 1 μg/mL of LPS for 24 h and 48 h did not cause any toxic effect. In this regard, literature data show that the exposition of ADSCs with this LPS dose provoked their activation. Specifically, it has been reported that the treatment of ADSCs with 1 μg/mL of LPS enhanced angiogenesis [118] and exerted hepatoprotective effects [119].

Noteworthy, a further increment of ADSC cell viability was observed in Cur-NCs-treated cells in both the presence and the absence of LPS compared to untreated cells, highlighting the protective effect of Cur-NCs on ADSCs grown at both the steady state or under inflammatory stimuli. These findings were confirmed by flow cytometry assay, which showed any variation in the cell population rates in LPS-treated ADSCs compared to control, at both time points. It is worth noting that the treatment of ADSCs with Cur alone (at 48 h) or combined with LPS (at both 24 h and 48 h) caused a slight but significant reduction in viable cells and a concomitant increment of late apoptotic cells compared to the control or LPS-treated cells. Conversely, the treatment with Cur-NCs did not cause any change in ADSC cell survival. In this regard, a plethora of studies showed the ability of the drug-delivery systems to improve the efficacy of Cur, compared to the free form of the natural compound [120,121,122,123].

Finally, considering the immunomodulatory activity of ADSCs, we also evaluated the ability of Cur-NCs to modulate the expression of the most common pro- and anti-inflammatory cytokines. Cytokines are small molecules crucial in regulating various cellular processes, including inflammation. In an inflammatory context, resident cells, such as macrophages, release a cascade of pro-inflammatory cytokines, including IL-1β and TNF-α, which act as signaling molecules, coordinating the host’s response to injury or infection [124]. Specifically, IL-1β and TNF-α play a central role in orchestrating the immune response by recruiting other immune cells to the inflammatory site or promoting the production of further pro-inflammatory cytokines [125]. The amplification of inflammatory response allows counteracting severe infections, by preventing pathogen growth and invasion. However, upon removing the causative agent, the inflammation should subside, thus allowing the so-called “*restitutio ad integrum*”. In this phase, the release of anti-inflammatory cytokines, such as IL-10 and TGF-β, allows dampening of the inflammatory response, thus promoting tissue repair and regeneration. In fact, these anti-inflammatory molecules can reduce the immune response to pathogens, thus preventing the harmful effects of chronic inflammation [126]. Indeed, failure in the resolution process determined a prolonged inflammatory state, predisposing to the development of various inflammatory-based diseases [7,127].

Considering that, our results showed a significant increase in the expression of the proinflammatory cytokines IL-1β and TNF-α and the anti-inflammatory cytokine IL-10 on ADSCs treated with LPS alone or in combination with other treatments, at both 24 h and 48 h, revealing the ability of this molecule to activate the inflammatory response. Furthermore, as expected the anti-inflammatory drug Dexa was able to reduce the expression of all tested cytokines, demonstrating its anti-inflammatory action. Interestingly, the expression of proinflammatory cytokines was significantly downregulated on ADSCs concomitantly treated with LPS and Cur-NCs, for 24 h and 48 h, compared to both control and LPS alone. Moreover, at both time points, the treatment with Cur-NCs induced a significant increase in the expression of anti-inflammatory cytokines IL-10 and TGF-β compared to the control and LPS. These findings support the hypothesis that PCL NCs can efficiently vehicle Cur inside the cells, thus promoting its anti-inflammatory and immunomodulatory action. As mentioned before, ADSCs possess immunomodulatory activity. Indeed, these cells secreted a variety of soluble factors, including anti-inflammatory cytokines (IL-10, TGF-β), growth factors, and extracellular vesicles, which can modulate immune responses. In our previous paper, we demonstrated the ability of both ADSCs and ADSCs differentiated in pericytes to modulate the expression of both pro- and anti-inflammatory cytokines proving a protective effect in an in vitro model of diabetic retinopathy [17,18,128]. On the other hand, overwhelming reports highlighted the anti-inflammatory and immunomodulatory activity of Cur [129,130,131,132,133]. Specifically, Cur demonstrated the ability to modulate the activity of immune cells, including T cells, B cells, dendritic cells, monocytes, macrophages, and neutrophils [133]. Asha et al. demonstrated the ability of Cur to inhibit T-cell proliferation and some inflammatory mediators such as interleukin-2, nitric oxide, and the transcription NF-kB. By inhibiting NF-kB target genes, Cur also reduces the production of cytokines, suppressing the immune response [134]. In this regard, an in vivo study demonstrated that inhibition of NF-kB induced by Cur determined a reduction in the pro-inflammatory cytokines IL-1β, IL-6, and TNF-α in the serum, counteracting fever in LPS-treated rabbits [135,136]. Furthermore, Cur also promotes the release of anti-inflammatory cytokines such as IL-4, IL-10, and IL-13 attenuating the inflammatory response [137]. Cur is also able to modulate the expression of TGF-β, playing a protective role in different pathological conditions [138]. However, Cur’s therapeutic potential is hindered by its poor water solubility, which results in scarce cellular uptake, rapid metabolism, and ultimately low bioavailability [133]. The development of Cur drug-delivery systems aimed to improve the bioavailability of Cur, and consequently, its biological properties, such as its anti-inflammatory and immunomodulatory activities, represent an active research area. Indeed, several studies demonstrated the ability of different nanocarriers to preserve the integrity of the natural compound, allowing it to exert its health-beneficial effects in different inflammatory contexts [139,140,141,142,143,144]. A plethora of in vivo studies demonstrate the beneficial effect of Cur in regulating the microbiota composition on both human and animal models [145,146,147,148,149]. In particular, Shen et al. showed that oral administration of Cur to C57BL/6 mice determined a variation in the abundance of several representative families in gut microbial communities, including Prevotellaceae, Bacteroidaceae, and Rikenellaceae, proving the beneficial effects of Cur at the intestinal level. However, the authors also reported that such effects should be less evident in humans due low bioavailability of the natural compound [150]. On the other hand, PCL nanoparticles seem to be a valid delivery system able to overcome the limits of therapeutic applications of Cur. In this regard, an interesting in vivo study showed the clinical application of Dexa-loaded PCL at the ocular level. Specifically, the authors demonstrated the feasibility and tolerance of intravitreous PCL drug delivery systems. Indeed, this system proved a controlled and prolonged delivery of Dexa in rabbit eyes, revealing a possible use of this drug delivery system for the treatment of intraocular diseases. A further study evaluated the toxicity of PCL degradation products on mouse mesenchymal stem cells from bone marrow. The authors used mesenchymal stem cells as a model system to mimic in vivo, demonstrating the absence of deleterious effects of PCL degradation products, thus proving its biocompatibility [151].

Thanks to its outstanding biological properties, ADSCs were considered a versatile tool in regenerative medicine. As mentioned earlier, ADSCs possess the ability to differentiate into various cell types, including adipocytes, osteoblasts, chondrocytes, myocytes, and even neurons. Given the ability of ADSCs to differentiate in chondrocytes, several studies explored the clinical application of these cells in the treatment of articular cartilage defects [152]. Such a study showed a potent chondrogenic potential of ADSCs, revealing their use in clinical tissue engineering. Besides this, cartilage defects, like osteoarthritis and other degenerative joint diseases, are usually comorbid with inflammation, immune dysfunction, and impairment in tissue regeneration. In this context, ADSCs have a dual advantage because they not only promote cartilage regeneration but also modulate the immune environment, an important feature for the reduction in inflammation and support for healing.

In this context, our findings highlighted the ability of Cur-NCs to improve the bioavailability of Cur and revealed the efficiency of this drug delivery system to vehicle the natural compound inside the ADSCs, thus promoting the immunomodulatory activity of human stem cells. Furthermore, at the intestinal level, Cur-NCs more efficiently inhibited pathogen growth (e.g., *E. coli*) without affecting probiotic bacteria’s survival, ensuring gut homeostasis. Taken together, our results shed light on the possible therapeutical applications of Cur-NCs. Specifically, by promoting the immunomodulatory activity of ADSCs, Cur-NCs could be useful in the cell-based treatment of inflammatory-driven diseases, such as osteoarthritis. Additionally, thanks to the action at the intestinal level Cur-NCs could be also used in the treatment of intestinal dysbiosis. However, further studies are needed to corroborate these hypotheses.

## 4. Materials and Methods

### 4.1. Chemicals and Reagents

Turmeric D.E. 95% curcuminoids (curcumin ≥ 70%) was purchased from Arda Natura s.r.l. (Fiorenzula D’Arda (PC), Italy); CCT was purchased from Farmalabor S.r.l. (Canosa di Puglia (BT), Italy); P80 was purchased from Fisher Chemical (Fisher Scientific, Geel, Belgium); PCL (Mn 45,000), SM, pepsin from porcine gastric mucosa, pancreatin from porcine pancreas, bile extract porcine, Dexa, and LPS from *E. coli* O111:B4 were purchased from Sigma-Aldrich (Milan, Italy); Water Chromasolv Plus for HPLC solvent was purchased from Honeywell Riedel-de-Haën (Seelze, Germany). Reagents for qPCR Trizol, High Capacity RNA-to-cDNA Kit, and Power SYBR^®^ Green PCR Master Mix, were purchased from Lifetechnologies™ (Foster-City, CA, USA).

### 4.2. Preparation of Curcumin-Nanocapsules

Cur-NCs were prepared according to the method described in the literature [52,53]. A stirring (300 rpm) solution at 40 °C of SM (76 mg), PCL (200 mg), CCT (300 mg), and Cur (20 mg) in an acetone/ethanol mixture (50 and 6 mL, respectively) was poured into a P80 (150 mg) solution in pure water (100 mL), and the resulting mixture was kept for 10 min at 40 °C. After volume reduction under vacuum (30 °C) to 20 mL, a yellow and lactescent nanosuspension of Cur-NCs was obtained. The pH value of the Cur-NCs suspension was measured by a SevenCompact pH Meter (Mettler Toledo, Milan, Italy) at 25 °C.

### 4.3. Characterization of Curcumin-Nanocapsules

#### 4.3.1. Particle Size and Zeta Potential

The Z-average, PDI, and intensity-weighted distribution (I-WD) of Cur-NC suspension were determined by DLS experiments. ζ value of Cur-NC suspension was determined by ELS experiments. The sample (10 µL) was diluted with 2 mL of pure water or pre-filtered (0.45 µm) 10 mM NaCl aqueous solution before DLS or ELS measurements, respectively. DLS and ELS experiments were performed on a Zetasizer Nano ZS-90 (Malvern Instruments, Malvern, UK) at 25 °C, and data were analyzed using Zetasizer Version 7.02 software.

#### 4.3.2. Encapsulation Efficiency of Curcumin-Nanocapsules

The total content of Cur in Cur-NCs suspension was determined following a protocol depicted by Granata et al. [52]. Briefly, the sample (50 µL) was treated with acetonitrile (1 mL) followed by ultrasonication (30 min) and centrifugation (30 min at 3500× *g*) by an Ultrasonic cleaner 600TH (VWR International bvba/sprl, Leuven, Belgium), frequency 45 kHz, power 1200 W, and Heraeus Pico 21 centrifuge (Thermo Scientific, Thermo Fisher, Waltham, MA, USA), respectively. An aliquot (100 µL) of supernatant was diluted with acetonitrile (400 µL) and analyzed by HPLC.

The HPLC analyses were performed on a Dionex HPLC system (P680 pump, ASI-100 autosampler, UVD170U detector, TCC-100 temperature-controlled column compartment, Dionex, Milan, Italy) and using Phenomenex Luna Omega (Phenomex S.r.l., Castel Maggiore, Bologna, Italy) 5 µm C18 reverse-phase column (150 × 4.6 mm). The mobile phase was composed of 2.5% formic acid in acetonitrile (A) and 2.5% formic acid in water (B). The linear gradient was A from 15% to 30% in 5 min, 30% A for 3 min, from 30% to 90% in 10 min, 90% A for 2 min, flow 1 mL/min, T = 40 °C, λ = 425 nm. For the quantitative determination of Cur, a calibration straight line (R^2^ = 0.9997) was previously constructed by means of eight solutions containing different Cur concentrations (from 14.0 to 111.6 µg/mL). The sample injection volume was 20 μL.

The free Cur was determined by the ultrafiltration/centrifugation technique (Nanosep 10K Omega, Pall Life Science, Milan, Italy; 90 min at 3500× *g*). The suspension (500 µL) of Cur-NCs was ultrafiltrated, and then the filtrate (50 µL) was diluted with acetonitrile. The resulting solution was analyzed by HPLC.

The EE was calculated using the following Equation (1):

(1) $$\mathrm{EE}\;\left(\%\right)=\frac{\left[\mathrm{Cur}\;\mathrm{encapsulated}\right]}{{\left[\mathrm{Cur}\right]}_{\mathrm{tot}}}\times 100$$

where the [Cur encapsulated] = [Cur]_tot_ − [Cur]_free_ represents the content of Cur encapsulated in Cur-NCs suspension; the [Cur]_tot_ and the [Cur]_free_ are the total and free content of Cur in Cur-NCs suspension, respectively.

#### 4.3.3. Ultraviolet–Visible and Fluorescence Spectra of Curcumin-Nanocapsules

The UV−vis and fluorescence spectra of Cur-NCs were recorded on an 8453 UV−Vis spectrophotometer (Agilent Technologies, Milan, Italy) and on a Fluoromax-3 fluorescence spectrometer (Horiba-Jobin-Yvon, Rome, Italy), respectively. To perform the UV−vis and fluorescence (λ_exc_ 420 nm, slits 2) spectra of Cur-NCs, 10 μL of the nanosuspension was diluted with 3 mL of pure water. About 10 μL of the Cur-NCs nanosuspension were added to 3 mL of acetonitrile to break the nanocapsules to record the spectrum of released Cur. Both fluorescence spectra (in water and acetonitrile) were obtained by subtracting the raw spectra from Cur_free_ (obtained by ultrafiltration/centrifugation technique) and NCs’ very low emissions (10 μL of each sample diluted with 3 mL of pure water or acetonitrile).

### 4.4. Stability of Curcumin-Nanocapsules over Time

The Cur-NCs nanosuspension was stored at two different temperatures (25 °C and 40 °C) for 30 days. Particle size distribution, PDI, ζ value, and Cur retention (%) were monitored over the storage time. Cur retention (%) was estimated by the following Equation (2):

(2) $$\mathrm{Cur}\;\mathrm{retention}\;\left(\%\right)=\frac{{\left[\mathrm{Curencapsulated}\right]}_{t}}{{\left[\mathrm{Curencapsulated}\right]}_{0}}\times 100$$

where the numerator is the Cur-encapsulated concentration at the time t and the denominator is the Cur-encapsulated concentration of freshly prepared suspensions, both were determined by HPLC as described in Section 4.3.2. All data are expressed as mean ± standard deviation (SD). The analysis of variance (ANOVA) followed by mean comparison (Tukey’s test) at a significance level of 0.05 was performed on the experimental stability data.

### 4.5. Simulated Gastrointestinal Digestion Test of Curcumin-Nanocapsules

#### 4.5.1. Gastric and Intestinal Phases

The simulated digestion assay was accomplished by adapting a protocol described by Granata et al. [55]. In particular, 1 mL of Cur-NCs suspension was added to 9 mL of simulated gastric fluid with porcine pepsin (30 mg), and the mixture (pH 1.5) was shaken (100 rpm, 37 °C). After the gastric digestion (2 h), 1 mL of the acidic mixture was withdrawn to perform DLS and ELS analyses. The remainder was treated with aqueous sodium bicarbonate (2.25 mL, 0.75 M) to bring the pH to 7.0. 5 mL of the simulated intestinal fluid, containing porcine pancreatin (17 mg) and porcine bile extract (77 mg), which were added to 5 mL of the (neutralized) gastric mixture. After incubation (100 rpm, 37 °C for 2 h), an aliquot (1 mL) was centrifuged for 10 min at 10,000× *g*, and 500 µL of MeOH was added to 500 µL of the supernatant (“micellar phase”). An aliquot of uncentrifuged samples was treated identically. All samples (gastric digest, methanol-treated raw intestinal digest, and micellar phase) were kept at −20 °C until HPLC analyses.

#### 4.5.2. Physicochemical Characterization of Curcumin-Nanocapsules after Simulated Gastric Digestion

The Z-average, PDI, I-WD, and ζ values of Cur-NCs were obtained by DLS or ELS experiments as above described. In particular, 100 µL of Cur-NCs gastric digest was diluted with 2000 µL of pure water or pre-filtered (0.45 µm) 10 mM NaCl aqueous solution for DLS or ELS, respectively. Both acidic and neutral (treated with sodium bicarbonate) digests were analyzed.

#### 4.5.3. Determination of Curcumin Concentration after Digestion Phases

Cur amount after digestion phases was estimated via HPLC analyses. Cur-encapsulated amount in the Cur-NCs after simulated gastric digestion was calculated as [Cur]_tot_ − [Cur]_free_ through a procedure similar to the one described in Section 4.3.2. In particular, to determine [Cur]_tot_ (150 µL) an aliquot of the sample after acidic was diluted with an equal volume (150 µL) of acetonitrile and centrifuged for 10 min at 10,000× *g*. 200 µL of supernatant diluted with 800 µL di acetonitrile, re-centrifuged (5 min at 3500× g), and, then, analyzed by HPLC. [Cur]_free_ was obtained by ultrafiltration/centrifugation method, followed by HPLC analysis as above described.

The B, S, and EB values of Cur were calculated using the following Equations (3)–(5) [41,55,59]:

(3) $$B\;\left(\%\right)=\frac{{\mathrm{Cur}}_{\mathrm{micelle}}}{{\mathrm{Cur}}_{\mathrm{digesta}}}\times 100$$

(4) $$S\;\left(\%\right)=\frac{{\mathrm{Cur}}_{\mathrm{micelle}}}{{\mathrm{Cur}}_{\mathrm{digesta}}}\times 100$$

(5) $$\mathrm{EB}\;\left(\%\right)=\frac{{\mathrm{Cur}}_{\mathrm{micelle}}}{{\mathrm{Cur}}_{\mathrm{digesta}}}\times 100$$

where Cur_micelle_, Cur_digesta_, and Cur_initial_ are the concentrations of Cur in the micelle fraction, in the raw digesta, and in the initial sample, respectively. For quantifying Cur_micelle_, Cur_digesta_, and Cur_initial_ 200 µL of each sample was diluted with 200 µL di methanol and centrifuged for 10 min at 10,000× *g*. About 200 µL of supernatant was added to 800 µL of acetonitrile, re-centrifuged (5 min at 3500× *g*), and analyzed by HPLC.

### 4.6. Microbiological Assay

The antibacterial activity of Cur, NCs, Cur-NCs, and Gen was determined by the microdilution method [60]. For the microbiological assay, six Gram-positive ATCC strains (*S. aureus* ATCC 6538, *E. faecalis* ATCC 29212, *L. delbrueckii* ATCC 11842, *Lactobacillus plantarum* (*L. plantarum*) ATCC BAA 793, *Lactobacillus reuteri* (*L. reuteri*) ATCC 23272, *Lactobacillus rhamnosus* (*L. rhamnosus*) GG ATCC 53103) and two Gram-negative ATCC strains (*E. coli* ATCC 8728, *P. aeruginosa* ATCC 9027) were used. Bacterial strains were purchased from LGC Limited (Teddington, Middlesex, UK). The antibacterial effect against *S. aureus*, *E. faecalis*, *E. coli*, and *P. aeruginosa* was evaluated according to the procedures of the CLSI M100-S30 [61]. The substances were diluted in the 1:100 ratio in BBLTM Cation-adjusted Mueller Hinton II Broth (CAMHB) (Becton Dickin-son, Franklin Lakes, NJ, USA). The stock solutions were filtered with 0.22 μm filters (MF-Millipore, Merck, Germany), and serial dilutions were obtained in 96-well microplates (Corning, New York, USA) in concentrations ranging from 0.97 to 500.00 μg/mL (Cur and Cur-NCs) and 0.25–128.00 μg/mL (Gen), respectively. Unloaded nanoparticles (NCs) represented the negative control. Bacterial colonies grown on Mueller Hinton Agar (MHA) plates (Oxoid, Milan, Italy) were picked and suspended in 0.9% saline obtaining turbidity equivalent to 0.5 McFarland standard (1.5 × 10^8^ colony forming units (CFU)/mL). The turbidity of the suspensions was checked by a spectrophotometer at λ = 600 nm (Bio-Tek Synergy HT Microplate Reader, Bio-Tek Instruments, Winooski, VT, USA). Bacterial suspensions were diluted in the 1:100 ratio in CAMHB and added to the wells, to a final concentration of 5 × 10^5^ CFU/mL. The microplates were incubated at 37 °C for 24 h. The antibacterial activity against *Lactobacillus* spp. was performed according to the guidelines of the CLSI M45-A2 [62]. Specifically, serial dilutions of the substances were obtained in CAMHB (Becton Dickin-son, USA) supplemented with 5% *v*/*v* lysed horse blood (bioMérieux, Marcy l’Etoile, France). *Lactobacillus* spp. colonies grown on De Man Sharp Rogosa Agar plates (Oxoid, Milan, Italy) after 24 h of incubation at 35° C under anaerobic conditions were picked and suspended in 0.9% saline, obtaining turbidity equivalent to 0.5 McFarland standard. The suspensions were diluted in the 1:100 ratio in CAMHB supplemented with 5% *v/v* lysed horse blood and added to the wells to a final concentration of 5 × 10^5^ CFU/mL. The microplates were incubated at 35 °C for 48 h in a 5% CO_2_ atmosphere. Each assay included a positive growth control and a negative sterility control.

### 4.7. Cell Culture

Adipose tissue was harvested from four healthy female donors (32–38 years old) undergoing liposuction procedures at the Cannizzaro Hospital (Catania, Italy). The donors were non-smokers and did not take estrogen replacement therapy. Lipoaspirate was obtained from the abdominal region after donors had signed an informed consent form for the use of lipoaspirate for experimental procedures, in accordance with the Declaration of Helsinki. The protocol was approved by the Local Ethics Committee (Ethic Committee Catania1; Authorization n. 398/2021/EMPO). The raw lipoaspirate (50–100 mL) was incubated for 3 h at 37 °C with an equal volume of serum-free low-glucose Dulbecco’s Modified Eagle’s Medium (DMEM; Sigma-Aldrich, Milan, Italy) containing 0.075% type I collagenase (Invitrogen, Monza, Italy). After the inactivation of collagenase activity by adding an equal volume of DMEM containing 10% heat-inactivated FBS (Gibco, Monza, Italy), the digested lipoaspirate was centrifuged at 1200 rpm for 10 min. The pellets were then washed in phosphate-buffered saline (PBS; Invitrogen), filtered through a 100 µm nylon cell strainer (Falcon BD Biosciences, Milan, Italy), and the cells were plated in T75 culture flasks (Falcon BD Biosciences) with DMEM containing 10% FBS, 1% P/S solution, and 1% MSC growth supplement (ScienCell Research Laboratories, Milan, Italy). After 24 h incubation at 37 °C with 5% CO_2_, non-adherent cells were removed by replacing the growth medium. When reaching confluence, all cultures were expanded for 2–3 passages and plated for the subsequent procedures. Some cell samples were used to verify their MSC nature, according to procedures previously described [17]. In particular, their positivity for typical MSC markers (CD44, CD73, CD90, and CD105) was confirmed by immunocytochemistry and flow cytometry, and their negative immune response for typical hematopoietic stem cell markers (CD14, CD34, and CD45) was verified.

### 4.8. Cell Culture Treatments and MTT Assay

To verify the possible cytotoxic effect of NCs, empty or loaded with Cur, on ADSCs, the MTT assay was used (Sigma-Aldrich, Milan, Italy). Cells were seeded in 96-well plates at the density of 1.5 × 10^4^ cells per well and incubated overnight at 37 °C, 5% CO_2_ in a humidified atmosphere before experiments. After this period, cells were treated with increasing concentrations (0.06, 0.125, 0.25, 0.5, 1 μg/mL) of Cur or Cur-NCs, for 24 h and 48 h. The cytotoxicity of NCs was analyzed by diluting them with the same dilution ratios used for Cur and Cur-NCs. Specifically, starting from a stock solution of 1 mg/mL of Cur or Cur-NCs, we prepared a working standard solution (WSS) from which we performed serial dilutions in the 1:2 ratio to obtain the tested concentrations. Analogously, the stock solution of NCs, prepared with the same procedure as Cur-NCs, was diluted to obtain the WSS, which corresponds to the concentration of 1 μg/mL of Cur and Cur-NCs. The WSS was then diluted in the 1:2; 1:4, 1:8, and 1:16 ratios as was performed for Cur and Cur-NCs solutions. LPS (500 μg/mL) was dissolved in a cell culture medium to obtain a WSS, which was further diluted to a specific concentration (1 μg/mL) for cell treatments. The stock solution of Dexa (10 nM) was dissolved with cell culture medium, obtaining a WSS which was further diluted to the tested concentration (10 nM).

The combined treatments were obtained by mixing a 2× concentration with respect to the final concentration of each solution (Cur 0.25 μg/mL+ LPS 2 μg/mL; NCs 1:4 + LPS 2 μg/mL; Cur-NCs 0.25 μg/mL + LPS 2 μg/mL and Dexa 20 nM + LPS 2 μg/mL).

A further MTT test was performed on ADSCs to evaluate the potential cytotoxic effect of the following treatments: 0.125 μg/mL Cur, 0.125 μg/mL Cur-NCs, NCs diluted in the 1:8 ratio, 10 nM Dexa with or without 1 µg/mL LPS, at 24 h and 48 h (Figure 6). In both cases, at the end of each time point, cells were incubated with an MTT solution (5 mg/mL) for 3 h at 37 °C. Next, 100 μL dimethyl sulfoxide was dispensed in each well, and plates were shaken for 10 min, to dissolve the formazan crystals formed during the incubation. The absorbance was measured at 570 nm with a plate reader (Synergy 2-bioTek, Bio-Tek Instruments, Winooski, VT, USA).

### 4.9. Muse Assay

The apoptotic and necrotic cell death in our experimental conditions was evaluated using the Muse^®^ Cell Analyzer (S/N: 7200121367, Merck, Darmstadt, Germany), as previously reported [153]. Briefly, ADSCs were seeded in 24-well plates at the density of 1 × 10^5^ cells per well and incubated overnight at 37 °C under a humidified atmosphere of 5% CO_2_, for 24 h and 48 h. After the incubation period, cells were washed twice with PBS, trypsinized, and resuspended in Annexin V & Dead Cell Reagent (Muse^®^ Annexin V & Dead Cell Kit Luminex Corporation, Merck, Darmstadt, Germany) according to the manufacturer’s instructions. Based on their staining, cells were distinguished into four cell populations: viable cells [Annexin V-PE (−) and 7AAD (−)], early apoptotic cells [Annexin VPE (+) and 7AAD (−)], late apoptotic cells [Annexin V-PE (+) and 7AAD (+)], and dead cells [Annexin V-PE (−) and 7AAD (+)]. The distribution of acquired events in the scatter plots is shown in the Results section.

### 4.10. Real-Time PCR

Total RNA was extracted using Trizol^®^ reagent (Invitrogen, Carlsbad, CA, USA) according to the manufacturer’s instructions. cDNA synthesis was performed with the High-Capacity cDNA Reverse Transcription kit (Applied Biosystems, Foster City, CA, USA). Real-time quantitative PCR (RT-qPCR) was conducted using SYBR Green PCR MasterMix (Life Technologies, Monza, Italy) on a Step-One Fast Real-Time PCR system (Applied Biosystems). Primer sequences of the studied genes were obtained from Metabion International AG (Planneg, Germany) and are listed below (Table 5).

### 4.11. Statistical Analysis

Data are presented as mean ± standard deviation (SD) from three independent experiments performed in triplicate. Statistical analyses were conducted using one-way or two-way ANOVA as specified in figure legends. Data were analyzed and visualized using GraphPad Prism 8 software.

## 5. Conclusions

Our results demonstrated that PCL NCs are stable and efficient green nanosystems able to effectively protect Cur from gastrointestinal degradation. Cur-loaded PCL nanocapsules (Cur-NCs) significantly enhance the bioavailability and therapeutic efficacy of Cur. By improving stability during gastrointestinal digestion and selectively inhibiting pathogenic bacteria while preserving beneficial gut microbiota, Cur-NCs offer a promising approach to the treatment of inflammatory diseases and intestinal dysbiosis. Additionally, Cur-NCs potentiate the immunomodulatory effects of ADSCs, highlighting their potential in cell-based therapies for inflammation-driven conditions.

## Figures and Tables

**Table 1:** Stability over time of curcumin-nanocapsules at 25 °C.

Storage Time (Days)	0	7	15	21	30
Z-average diameter (nm)	223 ± 13 ^a^	222 ± 14 ^a^	224 ± 16 ^a^	221 ± 11 ^a^	226 ± 15 ^a^
PDI	0.09 ± 0.03 ^a^	0.07 ± 0.02 ^a^	0.07 ± 0.03 ^a^	0.06 ± 0.02 ^a^	0.05 ± 0.02 ^a^
ζ (mV)	−17 ± 3 ^a^	−16 ± 4 ^a^	−16 ± 1 ^a^	−17 ± 2 ^a^	−15 ± 3 ^a^
Cur encapsulated amount (mg/mL)	1.00 ± 0.02 ^a^	1.00 ± 0.07 ^a^	1.00 ± 0.06 ^a^	1.00 ± 0.07 ^a^	0.97 ± 0.05 ^a^

Values in the same line with the same superscripts are not significantly different (*p* > 0.05).

**Table 2:** Stability over time of curcumin-nanocapsules at 40 °C.

Storage Time (Days)	0	7	15	21	30
Z-average diameter (nm)	223 ± 13 ^a^	218 ± 14 ^a^	222 ± 13 ^a^	220 ± 14 ^a^	223 ± 14 ^a^
PDI	0.09 ± 0.03 ^a^	0.07 ± 0.04 ^a^	0.07 ± 0.03 ^a^	0.05 ± 0.01 ^a^	0.05 ± 0.03 ^a^
ζ (mV)	−17 ± 3 ^a^	−17 ± 4 ^a^	−19 ± 1 ^a^	−18 ± 2 ^a^	−17 ± 2 ^a^
Cur encapsulated amount (mg/mL)	1.00 ± 0.02 ^a^	1.00 ± 0.07 ^a^	1.00 ± 0.04 ^a^	0.93 ± 0.04 ^a^	0.75 ± 0.06 ^b^

Values in the same line with the same superscripts are not significantly different (*p* > 0.05).

**Table 3:** Physicochemical parameters of curcumin-nanocapsules (Cur-NCs) after simulated gastric digestion (SGD) test.

Cur-NCs after SGD Test	pH 1.5	pH 7.0
Z-average diameter (nm)	210 ± 5	204 ± 4
PDI	0.09 ± 0.04	0.07 ± 0.04
ζ (mV)	−3 ± 1	−24 ± 1

**Table 4:** Antibacterial activity of empty nanocapsules (NCs), curcumin (Cur), curcumin-nanocapsules (Cur-NCs), and gentamycin (Gen) against Gram-positive and Gram-negative strains.

	MIC ^1^ (µg/mL)				
	NCs ^2^	Cur ^3^	Cur-NCs ^4^	Gen ^5^	
Bacterial Strains		[0.97–500.00]	[0.97–500.00]	[0.25–128.00]	I.C. ^6^
Gram-positive					
*Staphylococcus aureus* ATCC 6538	n.e. ^7^	62.50	500.00	0.50	S
*Enterococcus faecalis* ATCC 29212	n.e.	7.81	>500.00	32.00	n.r. ^8^
*Lactobacillus delbrueckii* ATCC 11842	n.e.	250.00	>500.00	8.00	I
*Lactobacillus plantarum* ATCC BAA 793	n.e.	>500.00	>500.00	>128.00	R
*Lactobacillus reuteri* ATCC 23272	n.e.	>500.00	>500.00	128.00	R
*Lactobacillus rhamnosus* GG ATCC 53103	n.e.	>500.00	>500.00	128.00	R
Gram-negative					
*Escherichia coli* ATCC 8728	n.e.	62.50	31.25	2.00	S
*Pseudomonas aeruginosa* ATCC 9027	n.e.	250.00	>500.00	0.50	S

^1^ MIC: minimum inhibitory concentration; ^2^ NCs: empty nanocapsules; ^3^ Cur: curcumin; ^4^ Cur-NCs: curcumin-loaded nanocapsules; ^5^ Gen: gentamycin; ^6^ I.C.: interpretive criteria for gentamycin (CLSI M100-S30): *Staphylococcus aureus*, *Escherichia coli*, *Pseudomonas aeruginosa* ≤ 4 susceptible (S), 8 intermediate (I), ≥16 resistant (R); interpretive criteria for gentamycin (CLSI M45-A2): *Lactobacillus* spp., ≤4 susceptible (S), 8 intermediate (I), ≥16 resistant (R); ^7^ n.e.: no effect; ^8^ n.r.: interpretive criteria for gentamycin not reported in CLSI M100-S30.

**Table 5:** Primer sequences of the studied genes.

Gene Symbol	Forward	Reverse
IL-1β ^1^	AGCTCGCCAGTGAAATGATG	GTCGGAGATTCGTAGCTGGA
TGF-β ^2^	CAATTCCTGGCGATACCTCAG	GCACAACTCCGGTGACATCAA
TNF-α ^3^	GCAACAAGACCACCACTTCG	GATCAAAGCTGTAGGCCCCA
IL-10 ^4^	GACTTTAAGGGTTACCTGGGTTG	TCACATGCGCCTTGATGTCTG
β-actin	ACGTTGCTATCCAGGCTGTGCTAT	TTAATGTCACGCACGATTTCCCGC

^1^ IL-1β: interleukin-1β; ^2^ TGF-β: transforming growth factor-β; ^3^ TNF-α: tumor necrosis factor-α; ^4^ IL-10: interleukin-10.

## Data Availability

The data presented in this study are available on request from the corresponding author.
